# Assessing Capacity to Consent in Older Adults with Cognitive Impairment: Recommendations for Repeated Assessment in a Two‐Year, Internet‐Based Clinical Trial

**DOI:** 10.1002/alz70861_108142

**Published:** 2025-12-23

**Authors:** Meghan K Mattos, Kirsten MacDonnell, Jennifer H Lingler, Lee M Ritterband, Wen You, Carol A Manning

**Affiliations:** ^1^ University of Virginia, Charlottesville, VA USA; ^2^ University of Pittsburgh Alzheimer's Disease Research Center (ADRC), Pittsburgh, PA USA; ^3^ University of Pittsburgh, Pittsburgh, PA USA

## Abstract

**Background:**

Ongoing capacity‐to‐consent (CTC) assessments are essential in long‐term studies with cognitively impaired adults to protect research participants, uphold ethical research standards, and ensure the validity of study findings. This study describes the development, implementation, and benefits of repeated CTC assessments in a two‐year, Internet‐based randomized controlled trial (RCT) involving older adults living with cognitive concerns.

**Method:**

*SHUTi MIND* is an RCT evaluating an online insomnia intervention in older adults with cognitive concerns, using rolling recruitment. CTC is assessed via phone at baseline and every six months (6, 12, 18, and 24 months) using a standardized, IRB‐approved protocol. The protocol was developed based on current literature, study‐specific considerations, and input from neuropsychologists. A trained staff member conducts semi‐structured interviews that include (1) assessing participants’ understanding of recent, past, and upcoming events and (2) reviewing and evaluating their comprehension of the study’s purpose, risks, benefits, voluntariness, and decision‐making rationale. Up to three phone calls and three emails are made. If participants cannot demonstrate CTC, they are deemed to lack capacity, and surrogate consent is attempted. CTC completion rates and contact attempts were analyzed at the 6‐ and 12‐month assessments.

**Result:**

Of the total study participants (*N* =176), 80 had enrolled for at least 6 months and were eligible for the 6‐month CTC assessment. Sixty‐one participants (76.25%) completed the 6‐month CTC assessment, with 183 total contact attempts (mean = 2.29 contacts/participant). At 12 months, 43 were eligible for the 12‐month CTC check, and 32 (74.42%) completed the CTC assessment with 103 contact attempts (mean = 2.40 contacts/participant). One participant was determined to lack CTC, and surrogate consent was successfully obtained.

**Conclusion:**

The structured CTC protocol, including proactive follow‐up by phone and email, yielded high completion rates and allowed for consistent monitoring of decisional capacity over time. These findings suggest that routine CTC assessments in remote clinical trials involving cognitively impaired participants are both feasible and effective for identifying participants who may require additional ethical safeguards.

